# Supplementary material for: Adverse events following immunization with ChAdOx1 nCoV-19 and BBIBP-CorV vaccine: A comparative study among healthcare professionals of Nepal
Source: PLoS One. 2022 Aug 10;17(8):e0272729. doi: 10.1371/journal.pone.0272729 (PMC9365127; doi:10.1371/journal.pone.0272729)
Supplement: Attachment — Submitted filename: PONE-D-21-28369_HZ.pdf [file pone.0272729.s002.pdf]

PLOS ONE

Adverse events following immunization with ChAdOx1 nCoV-19 and BBIBP-CorV vaccine: a real-world, comparative study among healthcare professionals of Nepal

--Manuscript Draft--

**Manuscript Number:**	PONE-D-21-28369
**Article Type:**	Research Article
**Full Title:**	Adverse events following immunization with ChAdOx1 nCoV-19 and BBIBP-CorV vaccine: a real-world, comparative study among healthcare professionals of Nepal
**Short Title:**	ChAdOx1 nCoV-19 and BBIBP-CorV vaccine
**Corresponding Author:**	Md. Golam Hossain<br>University of Rajshahi<br>Rajshahi, BANGLADESH
**Keywords:**	: Adverse events following immunization; ChAdOx1 nCoV-19 vaccine; BBIBP-CorV vaccine; COVID-19
**Abstract:**	Background: Adverse events following immunization (AEFI) against SARS-CoV-2 are common as reported by clinical trials and contemporary evidence. The objective of the present study was to evaluate the local and systemic adverse events following vaccination with ChAdOx1 nCoV-19 and BBIBP-CorV among the healthcare professionals (HCPs) of Nepal.<br>Methods: This cross-sectional study was conducted among 606 vaccinated HCPs of Kathmandu, Nepal in between June 15 to 30, 2021 using a self-administered online survey tool. Multiple binary logistic regression models were used to predict the adverse events according to the vaccine types and doses after adjusting for age, sex, comorbidity, and previous SARS-CoV-2 infection.<br>Results: The mean (SD) age of the participants was 35.6 (13.2) years and 52% of them were female. Almost 59% of them were fully vaccinated and around 54% of total participants took the ChAdOx1 nCoV-19 vaccine. At least one local and systemic adverse event was reported by 54% and 62% participants after the first dose and 37% and 49% participants after the second dose of ChAdOx1 nCoV-19 and by 37% and 43% after the first dose and 42% and 36% after the second dose of BBIBP-CorV vaccine respectively. Injection site pain, swelling, and tenderness at the injection site were the most frequently reported local AEFI while, fatigue, headache, fever, and myalgia were the most frequently reported systemic AEFI. The logistic model demonstrated that the risk of both local and systemic adverse events was higher among the ChAdOx1 nCoV-19 vaccine recipients compared to the BBIBP-CorV vaccine. Almost 10% of individuals reported a post-vaccination SARS-CoV-2 infection and most of them occurred after taking the first dose of vaccine.<br>Conclusions: Most of the HCPs of Nepal experienced only mild and constitutional symptoms after vaccination. The target population of the mass vaccination campaign should be well assured about the adverse events with such evidence to make them confident in vaccine uptake.
**Order of Authors:**	Sushil Rayamajhi
	Md. Abdur Rafi
	Nishant Tripathi
	Anjana Singh Dong
	Minalma Pandey
	Shreejana Rayamajh
	Pranay Shrestha
	Md. Golam Hossain
	M Tasdik Hasan
**Additional Information:**	

Powered by Editorial Manager® and ProduXion Manager® from Aries Systems Corporation

Question	Response
**Financial Disclosure**<br><br>Enter a financial disclosure statement that describes the sources of funding for the work included in this submission. Review the submission guidelines for detailed requirements. View published research articles from *PLOS ONE* for specific examples.<br><br>This statement is required for submission and **will appear in the published article** if the submission is accepted. Please make sure it is accurate.<br><br>**Unfunded studies**<br>Enter: *The author(s) received no specific funding for this work.*<br><br>**Funded studies**<br>Enter a statement with the following details:<br>• Initials of the authors who received each award<br>• Grant numbers awarded to each author<br>• The full name of each funder<br>• URL of each funder website<br>• Did the sponsors or funders play any role in the study design, data collection and analysis, decision to publish, or preparation of the manuscript?<br>• **NO** - Include this sentence at the end of your statement: *The funders had no role in study design, data collection and analysis, decision to publish, or preparation of the manuscript.*<br>• **YES** - Specify the role(s) played.<br><br>* typeset	The funders had no role in study design, data collection and analysis, decision to publish, or preparation of the manuscript.
**Competing Interests**<br><br>Use the instructions below to enter a competing interest statement for this submission. On behalf of all authors, disclose any competing interests that could be perceived to bias this work—acknowledging all financial support and any other relevant financial or non-financial competing interests.	The authors have declared that no competing interests exist.

This statement is required for submission and **will appear in the published article** if the submission is accepted. Please make sure it is accurate and that any funding sources listed in your Funding Information later in the submission form are also declared in your Financial Disclosure statement.<br><br>View published research articles from *PLOS ONE* for specific examples.<br><br>**NO authors have competing interests**<br><br>Enter: *The authors have declared that no competing interests exist.*<br><br>**Authors with competing interests**<br><br>Enter competing interest details beginning with this statement:<br><br>*I have read the journal's policy and the authors of this manuscript have the following competing interests: [insert competing interests here]*<br><br>* typeset	
**Ethics Statement**<br><br>Enter an ethics statement for this submission. This statement is required if the study involved:<br><br>• Human participants<br>• Human specimens or tissue<br>• Vertebrate animals or cephalopods<br>• Vertebrate embryos or tissues<br>• Field research<br><br>Write "N/A" if the submission does not require an ethics statement.<br><br>General guidance is provided below. Consult the submission guidelines for detailed instructions. **Make sure that all information entered here is included in the Methods section of the manuscript.**	The study was conducted in compliance with the Declaration of Helsinki, the International Conference on Harmonisation Guideline for Good Clinical Practice, and all applicable local regulations. The study protocol and informed consent were reviewed and approved by the Nepal Health Research Council (Ref: NHRC-461/2021).

Powered by Editorial Manager® and ProduXion Manager® from Aries Systems Corporation

**Format for specific study types**<br><br>**Human Subject Research (involving human participants and/or tissue)**<br>• Give the name of the institutional review board or ethics committee that approved the study<br>• Include the approval number and/or a statement indicating approval of this research<br>• Indicate the form of consent obtained (written/oral) or the reason that consent was not obtained (e.g. the data were analyzed anonymously)<br><br>**Animal Research (involving vertebrate animals, embryos or tissues)**<br>• Provide the name of the Institutional Animal Care and Use Committee (IACUC) or other relevant ethics board that reviewed the study protocol, and indicate whether they approved this research or granted a formal waiver of ethical approval<br>• Include an approval number if one was obtained<br>• If the study involved *non-human primates*, add *additional details* about animal welfare and steps taken to ameliorate suffering<br>• If anesthesia, euthanasia, or any kind of animal sacrifice is part of the study, include briefly which substances and/or methods were applied<br><br>**Field Research**<br><br>Include the following details if this study involves the collection of plant, animal, or other materials from a natural setting:<br>• Field permit number<br>• Name of the institution or relevant body that granted permission	
**Data Availability**<br><br>Authors are required to make all data underlying the findings described fully available, without restriction, and from the time of publication. PLOS allows rare exceptions to address legal and ethical concerns. See the PLOS Data Policy and FAQ for detailed information.	Yes - all data are fully available without restriction

A Data Availability Statement describing where the data can be found is required at submission. Your answers to this question constitute the Data Availability Statement and **will be published in the article**, if accepted.<br><br>**Important:** Stating 'data available on request from the author' is not sufficient. If your data are only available upon request, select 'No' for the first question and explain your exceptional situation in the text box.<br><br>Do the authors confirm that all data underlying the findings described in their manuscript are fully available without restriction?	
**Describe where the data may be found in full sentences. If you are copying our sample text, replace any instances of XXX with the appropriate details.**<br><br>• If the data are **held or will be held in a public repository**, include URLs, accession numbers or DOIs. If this information will only be available after acceptance, indicate this by ticking the box below. For example: *All XXX files are available from the XXX database (accession number(s) XXX, XXX.*).<br>• If the data are all contained **within the manuscript and/or Supporting Information files**, enter the following: *All relevant data are within the manuscript and its Supporting Information files.*<br>• If neither of these applies but you are able to provide **details of access elsewhere**, with or without limitations, please do so. For example:<br><br>*Data cannot be shared publicly because of [XXX]. Data are available from the XXX Institutional Data Access / Ethics Committee (contact via XXX) for researchers who meet the criteria for access to confidential data.*<br><br>*The data underlying the results presented in the study are available*	Data will be available on request to the corresponding author.

from (include the name of the third party and contact information or URL).<br>• This text is appropriate if the data are owned by a third party and authors do not have permission to share the data.<br><br>* typeset	
Additional data availability information:	

Manuscript Click here to access/download;Manuscript;Manuscript.docx

Title: Adverse events following immunization with ChAdOx1 nCoV-19 and BBIBP-CorV vaccine: a real-world, comparative study among healthcare professionals of Nepal

Authors:

Sushil Rayamajhi[a], Email: dr.sushil.rayamajhi@gmail.com

Md. Abdur Rafi[b,c], Email: abdurrafi007@gmail.com

Nishant Tripathi[d], Email: nishant55@icloud.com

Anjana Singh Dongol[e], Email: anjanadongol@yahoo.com

Minalma Pandey[a], Email: minalmapandey@hotmail.com

Shreejana Rayamajhi[f], Email: rshreezana@gmail.com

Subhechchha Bhandari[g], Email: subhechchha2018@teachfornepal.org

Pranay Shrestha[h], Email: pranay2018@teachfornepal.org

M Tasdik Hasan[i,j,k], Email: tasdikhdip@yahoo.com

Md. Golam Hossain[l], Email: hossain95@yahoo.com

Affiliations:

[a]Swacon International Hospital, Kathmandu, Nepal

[b]Rajshahi Medical College, Rajshahi, Bangladesh

[c]Pi Research Consultancy Center, Dhaka, Bangladesh

[d]Department of Medicine, University of Kentucky Medical Center, Kentucky, USA

[e]Kathmandu University School of Medical Science, Dhulikhel, Nepal

[f]Department of Anesthetia, Klinikum Ingolstadt Hospital, Ingolstadt, Germany

[g]Department of International Community Health, University of Oslo, Norway

[h]Department of Information Management, Tribhuvan University, Nepal

[i]Jeeon Bangladesh Ltd., Dhaka, Bangladesh

[j]Public Health Foundation, Bangladesh (PHF, BD), Dhaka 1217, Bangladesh

[k]Department of Primary Care & Mental Health, University of Liverpool, Liverpool L69 3BX, UK

[l]Health Research Group, Department of Statistics, University of Rajshahi, Rajshahi 6205, Bangladesh

Corresponding author

Md. Golam Hossain

Professor of Health Research Group

Department of Statistics

University of Rajshahi, Rajshahi 6205, Bangladesh

Email: hossain95@yahoo.com

Short title: ChAdOx1 nCoV-19 and BBIBP-CorV vaccine

Abstract

Background: Adverse events following immunization (AEFI) against SARS-CoV-2 are common as reported by clinical trials and contemporary evidence. The objective of the present study was to evaluate the local and systemic adverse events following vaccination with ChAdOx1 nCoV-19 and BBIBP-CorV among the healthcare professionals (HCPs) of Nepal.

Methods: This cross-sectional study was conducted among 606 vaccinated HCPs of Kathmandu, Nepal in between June 15 to 30, 2021 using a self-administered online survey tool. Multiple binary logistic regression models were used to predict the adverse events according to the vaccine types and doses after adjusting for age, sex, comorbidity, and previous SARS-CoV-2 infection.

Results: The mean (SD) age of the participants was 35.6 (13.2) years and 52% of them were female. Almost 59% of them were fully vaccinated and around 54% of total participants took the ChAdOx1 nCoV-19 vaccine. At least one local and systemic adverse event was reported by 54% and 62% participants after the first dose and 37% and 49% participants after the second dose of ChAdOx1 nCoV-19 and by 37% and 43% after the first dose and 42% and 36% after the second dose of BBIBP-CorV vaccine respectively. Injection site pain, swelling, and tenderness at the injection site were the most frequently reported local AEFI while, fatigue, headache, fever, and myalgia were the most frequently reported systemic AEFI. The logistic model demonstrated that the risk of both local and systemic adverse events was higher among the ChAdOx1 nCoV-19 vaccine recipients compared to the BBIBP-CorV vaccine. Almost 10% of individuals reported a post-vaccination SARS-CoV-2 infection and most of them occurred after taking the first dose of vaccine.

Conclusions: Most of the HCPs of Nepal experienced only mild and constitutional symptoms after vaccination. The target population of the mass vaccination campaign should be well assured about the adverse events with such evidence to make them confident in vaccine uptake.

Keywords: Adverse events following immunization; ChAdOx1 nCoV-19 vaccine; BBIBP-CorV vaccine; COVID-19.

Introduction

The world is currently facing the largest pandemic of the century due to a newly emerged highly transmissible and pathogenic virus of the coronavirus family, the SARS-CoV-2 which causes an acute respiratory disease, named 'coronavirus disease 2019' (COVID-19) (1). This pandemic contributed to more than 205 million infections by the virus and four million deaths worldwide (2). Vaccination against SARS-CoV-2 is recognized as a leading strategy to combat the pandemic and several vaccines have been promptly developed and approved by different health authorities for mass vaccination worldwide (3).

In Nepal, almost 721 thousand COVID-19 cases were reported with almost 10000 deaths by June 2021 (2). The government of Nepal has started a public vaccination program in late January 2021 and administered more than 4.5 million doses of ChAdOx1 nCoV-19 and BBIBP-CorV vaccine by June 2021 covering 8% of their total population (4). The ChAdOx1 nCoV-19 is an adenoviral vector vaccine manufactured by the Oxford-AstraZeneca, UK and BBIBP-CorV is an inactivated SARS-CoV-2 vaccine manufactured by the Sinopharm, China (3). The large-scale clinical trials reported adequate safety profile and antibody responses by both of the vaccines (5–8).

The clinical trials, as well as real-world evidence, reported some adverse events following immunization (AEFI) with these vaccines though most of these were mild constitutional symptoms. A population-based study from the UK conducted among more than half a million recipients of BNT162b2 mRNA vaccine (Pfizer) or ChAdOx1 nCoV-19 recombinant vaccine (Oxford-AstraZeneca) reported that almost 22% individuals after taking BNT162b2 vaccine, and 34% individuals after taking the ChAdOx1 nCoV-19 vaccine developed at least one systemic symptom, mostly mild headache, fatigue, and fever. Local symptoms like injection site pain, tenderness, and swelling were reported by almost half of the recipients (9). Severe adverse events

like anaphylaxis or thromboembolism were very rare after vaccination against SARS-CoV-2 (10–12).

Despite the facts, fear of side effects remains one of the major contributors to the COVID-19 vaccine hesitancy worldwide (13,14). Reports on vaccine side effects from own community could make the people confident about the vaccine. Moreover, there is hardly any data on the real-world side effects of ChAdOx1 nCoV-19 and BBIBP-CorV vaccine among the South-East Asian population. Healthcare professionals, especially physicians, nurses, and medical technicians can be a suitable group for reporting the side effects of vaccination due to their professional knowledge and trust in the population. Hence, the present study aims to report the local and systemic adverse events experienced following immunization with these two major vaccines deployed among the healthcare professionals (HCPs) of Nepal.

Methods

Study design and participants: This cross-sectional study was conducted among the HCPs working within the geographical area of Nepal and who took at least one dose of the ChAdOx1 nCoV-19 or BBIBP-CorV vaccine between February 15 and May 31, 2021, from different vaccination centers of Nepal. The sample size was calculated using the following formula:

n= $\frac{z^2 p(1-p)}{d^2}$, where, p = prevalence of side effects after vaccination, and d = precision of error.

Menni et al. reported that 33·7% of the ChAdOx1 nCoV-19 vaccine recipients developed at least one side effect (9). Using this information the calculated sample size was 343. The convenience sampling method was used to include participants. An online survey tool was used for data collection. Five physicians, three nurses, and three medical technologists were recruited to

circulate the survey link among their professional network through social media (Facebook). The survey link was also posted in different closed groups of these professionals with a description of the aims of the study and a request to fill it up. Finally, a total of 606 HCPs (386 physicians and 238 other staff such as nurses and medical technicians) filled up the form completely.

Data collection procedure: An online survey using Google Forms created with the guidance of previously published articles (9,15,16) was used for data collection. It had three parts: (i) the socio-demographic characteristics of the participants, (ii) self-reported side effects after vaccination, and (iii) history of SARS-CoV-2 infection before or after vaccination. If SARS-CoV-2 RNA or antigen was detected on a respiratory specimen collected ≥14 days after completing the primary series of the vaccines, it was defined as a vaccine breakthrough infection (17).

Statistical Analysis: All statistical analyses were conducted using the STATA version 16.0. Frequency distribution with percentage was used to report the side effects experienced by the participants. The Chi-square test was used to determine the association between two categorical variables. Finally, multiple binary logistic regression adjusted for age, sex, comorbidity, and previous SARS-CoV-2 infection was used to predict the side effects according to the vaccine types and doses.

Results

A total of 606 HCPs (386 physicians and 238 supporting staff such as nurses and medical technicians) was considered as the sample in this study. Their mean (SD) age was 35.6 (13.2) years and almost 52% of them were female. More than 32% of the participants were infected by SARS-CoV-2 before vaccination. 17.66% of participants had comorbidity (such as type 2 diabetes mellitus, hypertension, chronic respiratory diseases like asthma and COPD, history of

cardiovascular diseases, etc.). It was observed that around 54% of participants took the ChAdOx1 nCoV-19 vaccine and 46% took the BBIBP-CorV vaccine. The type of vaccine was decided by the government authority based on availability. Almost 59% of them were fully vaccinated (took both doses). Chi-square test showed that age, profession, comorbidity of participants and type of vaccine was significantly ($p<0.01$) associated with number of dose vaccinated (Table 1).

Table 1: Socio-demographic characteristics of the participants (n = 606)

Characteristics	Total, N(%)	Partially vaccinated (first dose), Total = 250; N(%)	Fully vaccinated (both doses), Total = 356; N(%)	p-value
Age (years) (mean 35.63, SD 13.17)				
<55	515 (84.98)	188 (32.30)	327 (56.19)	<0.001
≥55	91 (15.02)	62 (68.13)	29 (31.87)	
Sex				
Male	290 (47.85)	126 (39.25)	164 (51.09)	0.293
Female	316 (52.15)	124 (35.23)	192 (54.55)	
Profession				
Physician	386 (63.70)	115 (29.11)	253 (64.05)	<0.001
Supporting stuffs	238 (39.27)	135 (48.56)	103 (37.05)	
Comorbidity				
Yes	107 (17.66)	62 (55.86)	45 (40.54)	<0.001

No	499 (82.34)	188 (33.45)	311 (55.34)	
Previous SARS-CoV-2 infection				
Yes	194 (32.01)	74 (36.10)	120 (58.54)	0.286
No	412 (67.99)	176 (37.61)	236 (50.43)	
Vaccine type				
ChAdOx1 nCoV-19	328 (54.13)	101 (30.79)	227 (69.21)	<0.001
BBIBP-CorV	278 (45.87)	149 (53.60)	129 (46.40)	

The overall prevalence of local adverse events was 45% and 44% and the prevalence of systemic adverse events was 54% and 44% after the first and second dose respectively. It was observed that both local and systemic adverse events were more prevalent after both first and second doses of ChAdOx1 nCoV-19 compared to BBIBP-CorV (54% vs 37% and 62% vs 43% respectively after the first dose and 45% vs 42% and 49% vs 36% respectively after the second dose). Besides, these adverse events occurred more frequently after the first dose than the second dose of both vaccines. Pain, swelling, and tenderness at the injection site were the most frequently reported local adverse events after both the first and second dose of the vaccines. On the other hand, fatigue, headache, fever, and myalgia were the most frequently reported systemic adverse events (Table 2). No serious and life-threatening adverse event like anaphylaxis was reported.

Table 2: Adverse events reported by the participants after vaccination (n=606)

Adverse events	ChAdOx1 nCoV-19				BBIBP-CorV			
	First dose		Second dose		First dose		Second dose	
	n	%	N	%	N	%	n	%

Local adverse events								
Any local adverse event	178	54.3	102	44.9	102	36.7	54	41.9
Pain at injection site	130	39.6	64	28.2	76	27.3	32	24.8
Swelling	64	19.5	36	15.9	36	12.9	14	10.9
Tenderness	46	14.0	47	20.7	24	8.6	22	17.1
Itch	8	2.4	0	0.0	6	2.2	0	0.0
Swollen armpit gland	3	0.9	0	0.0	4	1.4	0	0.0
Redness	22	6.7	18	7.9	10	3.6	14	10.9
Warmth	29	8.8	22	9.7	8	2.9	2	1.6
Systemic adverse events								
Any systemic adverse event	205	62.5	112	49.3	120	43.2	46	35.7
Headache	109	33.2	66	29.1	71	25.5	20	15.5
Fatigue	144	43.9	80	35.2	71	25.5	31	24.0
Chills and shivering	32	9.8	16	7.0	8	2.9	6	4.7
Diarrhea	10	3.0	4	1.8	5	1.8	0	0.0
Fever	108	32.9	80	35.2	55	19.8	23	17.8
Arthralgia	8	2.4	30	13.2	10	3.6	4	3.1
Myalgia	41	12.5	37	16.3	6	2.2	8	6.2
Nausea	22	6.7	14	6.2	14	5.0	2	1.6
Rash	0	0.0	4	1.8	0	0.0	0	0.0

Adverse events experienced by the participants after both vaccines were homogenous in different socio-demographic groups of participants (Supplementary figure). The logistic regression model showed that both local and systemic adverse events after the first dose of vaccination occurred at

a comparatively higher rate in the case of ChAdOx1 nCoV-19 vaccine (aOR 1.81, 95% CI 1.29-2.54 for local adverse events and aOR 2.32, 95% CI 1.65-3.26 for systemic adverse events) while this trend was observed for only systemic adverse events after the second dose (aOR 1.63, 95% CI 1.02-2.58). On the other hand, no significant difference was observed in the case of local adverse events of the ChAdOx1 nCoV-19 vaccine after the first and second dose, while systemic adverse events were more likely after the first dose of the vaccine (aOR 1.76, 95% CI 1.24-2.49). In the case of the BBIBP-CorV vaccine, both local and systemic adverse events were more likely after the first dose (aOR 2.61, 95% CI 1.57-4.35 for local and aOR 3.55, 95% CI 2.08-6.05 for systemic adverse events) (Figure 1).

A total of 61 participants were infected with SARS-CoV-2 after vaccination (infection rate 10%). Among them, 52 cases were reported after the first dose of vaccination (27 after the ChAdOx1 nCoV-19 vaccine and 25 after the BBIBP-CorV vaccine). On the other hand, all of the 9 cases were reported after the second dose of the ChAdOx1 nCoV-19 vaccine. The median of the window period between vaccination and infection after the first dose was 13 days (range 3 to 43 days) and between vaccination and infection after the second dose was 6 days (range 3 to 12 days) (Figure 2). No vaccine breakthrough infection was reported.

Discussion

In this study, we investigated the adverse events experienced by the HCPs of Nepal following the administration of the two COVID-19 vaccines that are in use in Nepal. These two vaccines are being used by many South Asian countries so the findings are very relevant and important in this mass vaccination phase. According to our findings, more than half of the participants experienced mild (to moderate) local and systemic adverse events after vaccination.

At least one local and systemic adverse event was reported by almost 54% and 62% participants after the first dose and 37% and 49% participants after the second dose of ChAdOx1 nCoV-19 vaccine respectively in our study. A large-scale community-based study from the UK reported that local and systemic side effects occurred in 59% and 34% of participants respectively after the first dose of the same vaccine (9). Though the prevalence of local adverse events was somewhat similar, that of systemic adverse events was comparatively higher in our study. However, it was lower than the expected rate of 88% as reported by a phase-3 trial of the vaccine (6). Prevalence of common local and systemic adverse events like injection site pain and tenderness, headache, fever, fatigue, etc. were reported lower among the British population (9), and comparatively higher Iraqi and Korean populations (15,16) than our study.

In the case of the BBIBP-CorV vaccine, local and systemic adverse events were reported by almost 37% and 43% respectively after the first dose and 42% and 36% respectively after the second dose. These findings were quite similar to the reports of the phase-2 trials of the vaccine, where almost one-third of the vaccine recipients experienced at least one adverse event (8,18). Injection site pain (22%) was the most common adverse event in the trial followed by fever (4%) and fatigue (3%) (18). Though the prevalence of these adverse events was higher among our study participants compared to the trials, real-world evidence from Iraq reported a higher prevalence of these symptoms among the BBIBP-CorV vaccine recipients (15).

Our study revealed that the odds of both local and systemic adverse events were higher among the ChAdOx1 nCoV-19 vaccine recipients compared to the BBIBP-CorV vaccine. Evidence of comparative prevalence of adverse events of these two vaccines is scanty. One study conducted in Iraq supported our findings (15). We could not establish any significant association between vaccine adverse events and the age or sex of the recipients, though prevalence was slightly

higher among females. A higher prevalence of adverse events among females was also reported by some studies, though the role of age remained ambiguous (9,15). Besides, we found a slightly higher prevalence of adverse events among previously infected participants similar to a large-scale study (9). It is hypothesized that this increased reactogenicity may be due to higher antibody titers and increased immunogenicity in previously infected individuals (19).

Among our participants, almost 10% reported a post-vaccination SARS-CoV-2 infection and most of them occurred after taking the first dose of vaccine. However, no vaccine breakthrough COVID-19 was reported. A recent study among Indian health care workers reported that almost 36% of the total SARS-CoV-2 infection was post-vaccination infection and most of these occur after the first dose (17). The Center for Disease Control of the USA (CDC), reported 3,729 vaccine breakthrough cases out of 144 million vaccinated people by June 2021 (20). Long-term follow-up is necessary to snap the actual picture of vaccine breakthrough infection among the population.

The main strength of this study lies in the fact that it provides a comparative picture of the side effects that occurred after taking the ChAdOx1 nCoV-19 and BBIBP-CorV vaccine among the HCPs of Nepal. Despite the fact, there are some limitations of the study. First of all, the study included only a small number of HCPs based on convenience sampling. This could potentially exclude a good number of individuals with severe side effects. Being a self-reported study, recall bias could not be rolled out. Besides, the post-vaccination infection may not be generalized for the overall population as we know that healthcare workers are tested more frequently even if they are asymptomatic. Finally, we could investigate only short-term side effects of the vaccine. A sustainable and long-term surveillance method in the general population is necessary to evaluate possible future adverse events and remedies.

Conclusions: Adverse events of both vaccines deployed in Nepal are mostly constitutional symptoms like injection site pain and tenderness, headache, fever, and fatigue, and mild in severity. Life-threatening adverse events following immunization like anaphylaxis are rare. The ChAdOx1 nCoV-19 vaccine recipients reported a slightly higher rate of adverse events compared to the BBIBP-CorV vaccine recipients. The finding should be widely disseminated among mass people to make them more confident in vaccine uptake and reduce vaccine hesitancy in general.

List of abbreviations: AEFI: Adverse events following immunization, HCP: Health care professional, WHO: World Health Organization, cOR: Crude odds ratio, aOR: Adjusted odds ratio, CI: Confidence interval.

Acknowledgment: The authors would like to thank all the participants for their cooperation in conducting the study.

References

1. Hu B, Guo H, Zhou P, Shi ZL. Characteristics of SARS-CoV-2 and COVID-19 [Internet]. Vol. 19, Nature Reviews Microbiology. Nature Research; 2021 [cited 2021 Jun 18]. p. 141–54. Available from: https://pubmed.ncbi.nlm.nih.gov/33024307/
2. Dong E, Du H, Gardner L. An interactive web-based dashboard to track COVID-19 in real time. Vol. 20, The Lancet Infectious Diseases. Lancet Publishing Group; 2020. p. 533–4.
3. Kaur SP, Gupta V. COVID-19 Vaccine: A comprehensive status report. Virus Res. 2020 Oct 15;288:198114.
4. WHO. Nepal story on COVID-19 vaccine deployment: a good start [Internet]. [cited 2021 Jul 25]. Available from: https://www.who.int/about/accountability/results/who-results-report-2020-mtr/country-story/2020/nepal-story-on-covid-19-vaccine-deployment-a-good-

start

5. Ramasamy MN, Minassian AM, Ewer KJ, Flaxman AL, Folegatti PM, Owens DR, et al. Safety and immunogenicity of ChAdOx1 nCoV-19 vaccine administered in a prime-boost regimen in young and old adults (COV002): a single-blind, randomised, controlled, phase 2/3 trial. Lancet [Internet]. 2020 Dec 19 [cited 2021 Jun 19];396(10267):1979–93. Available from: https://doi.org/10.1016/
6. Voysey M, Clemens SAC, Madhi SA, Weckx LY, Folegatti PM, Aley PK, et al. Safety and efficacy of the ChAdOx1 nCoV-19 vaccine (AZD1222) against SARS-CoV-2: an interim analysis of four randomised controlled trials in Brazil, South Africa, and the UK. Lancet [Internet]. 2021 Jan 9 [cited 2021 Jun 20];397(10269):99–111. Available from: https://doi.org/10.1016/
7. Xia S, Zhang Y, Wang Y, Wang H, Yang Y, Gao GF, et al. Safety and immunogenicity of an inactivated SARS-CoV-2 vaccine, BBIBP-CorV: a randomised, double-blind, placebo-controlled, phase 1/2 trial. Lancet Infect Dis [Internet]. 2021 Jan 1 [cited 2021 Jun 20];21(1):39–51. Available from: https://covid19.who.int/
8. Zhang Y, Zeng G, Pan H, Li C, Hu Y, Chu K, et al. Safety, tolerability, and immunogenicity of an inactivated SARS-CoV-2 vaccine in healthy adults aged 18–59 years: a randomised, double-blind, placebo-controlled, phase 1/2 clinical trial. Lancet Infect Dis. 2021 Feb 1;21(2):181–92.
9. Menni C, Klaser K, May A, Polidori L, Capdevila J, Louca P, et al. Vaccine side-effects and SARS-CoV-2 infection after vaccination in users of the COVID Symptom Study app in the UK: a prospective observational study. Lancet Infect Dis [Internet]. 2021 Jul 1 [cited 2021 Jul 25];21(7):939–49. Available from:

http://www.thelancet.com/article/S1473309921002243/fulltext

10. Shimabukuro TT, Cole M, Su JR. Reports of Anaphylaxis After Receipt of mRNA COVID-19 Vaccines in the US—December 14, 2020-January 18, 2021. JAMA [Internet]. 2021 Mar 16 [cited 2021 Jul 25];325(11):1101–2. Available from: https://jamanetwork.com/journals/jama/fullarticle/2776557

11. Mahase E. AstraZeneca vaccine: Blood clots are "extremely rare" and benefits outweigh risks, regulators conclude. BMJ [Internet]. 2021 Apr 8 [cited 2021 Jul 25];373:n931. Available from: https://www.bmj.com/content/373/bmj.n931

12. Lebedev L, Sapojnikov M, Wechsler A, Varadi-Levi R, Zamir D, Tobar A, et al. Minimal Change Disease Following the Pfizer-BioNTech COVID-19 Vaccine. Am J Kidney Dis. 2021 Jul 1;78(1):142–5.

13. Sallam M. COVID-19 Vaccine Hesitancy Worldwide: A Concise Systematic Review of Vaccine Acceptance Rates. Vaccines [Internet]. 2021 Feb 1 [cited 2021 Jul 25];9(2):1–15. Available from: /pmc/articles/PMC7920465/

14. Bono SA, Villela EF de M, Siau CS, Chen WS, Pengpid S, Hasan MT, et al. Factors Affecting COVID-19 Vaccine Acceptance: An International Survey among Low- and Middle-Income Countries. Vaccines 2021, Vol 9, Page 515 [Internet]. 2021 May 17 [cited 2021 Aug 6];9(5):515. Available from: https://www.mdpi.com/2076-393X/9/5/515/htm

15. Almufty HB, Mohammed SA, Abdullah AM, Merza MA. Potential adverse effects of COVID19 vaccines among Iraqi population; a comparison between the three available vaccines in Iraq; a retrospective cross-sectional study. Diabetes Metab Syndr Clin Res Rev [Internet]. 2021 Sep 1 [cited 2021 Jul 25];15(5):102207. Available from: https://linkinghub.elsevier.com/retrieve/pii/S1871402121002277

16. Jeon M, Kim J, Oh CE, Lee J-Y. Adverse Events Following Immunization Associated with Coronavirus Disease 2019 Vaccination Reported in the Mobile Vaccine Adverse Events Reporting System. J Korean Med Sci [Internet]. 2021 Apr 1 [cited 2021 Jul 26];36(17):1–8. Available from: https://doi.org/10.3346/jkms.2021.36.e114

17. Blachere NE, Hacisuleyman E, Darnell RB. Vaccine Breakthrough Infections with SARS-CoV-2 Variants. https://doi.org/101056/NEJMc2107808 [Internet]. 2021 Jun 2 [cited 2021 Jul 26]; Available from: https://www.nejm.org/doi/10.1056/NEJMc2107808

18. Xia S, Wang Y, Zhang W, Xie Z, You W, Huang L, et al. Safety and immunogenicity of an inactivated SARS-CoV-2 vaccine, BBIBP-CorV: a randomised, double-blind, placebo-controlled, phase 1/2 trial. Artic Lancet Infect Dis [Internet]. 2021 [cited 2021 Jul 26];21:39–51. Available from: https://covid19.who.int/

19. Krammer F, Srivastava K, Alshammary H, Amoako AA, Awawda MH, Beach KF, et al. Antibody Responses in Seropositive Persons after a Single Dose of SARS-CoV-2 mRNA Vaccine. https://doi.org/101056/NEJMc2101667 [Internet]. 2021 Mar 10 [cited 2021 Jul 26];384(14):1372–4. Available from: https://www.nejm.org/doi/10.1056/NEJMc2101667

20. COVID-19 Breakthrough Case Investigations and Reporting | CDC [Internet]. [cited 2021 Jun 19]. Available from: https://www.cdc.gov/vaccines/covid-19/health-departments/breakthrough-cases.html

Figure Legend

Figure 1: Risk of adverse events according to vaccine type and doses (OR with 95% CI adjusted for age, sex, comorbidities, and previous SARS-CoV-2 infection)

Figure 2: SARS-CoV-2 infection among the participants up to 8 weeks after vaccination (n = 61).

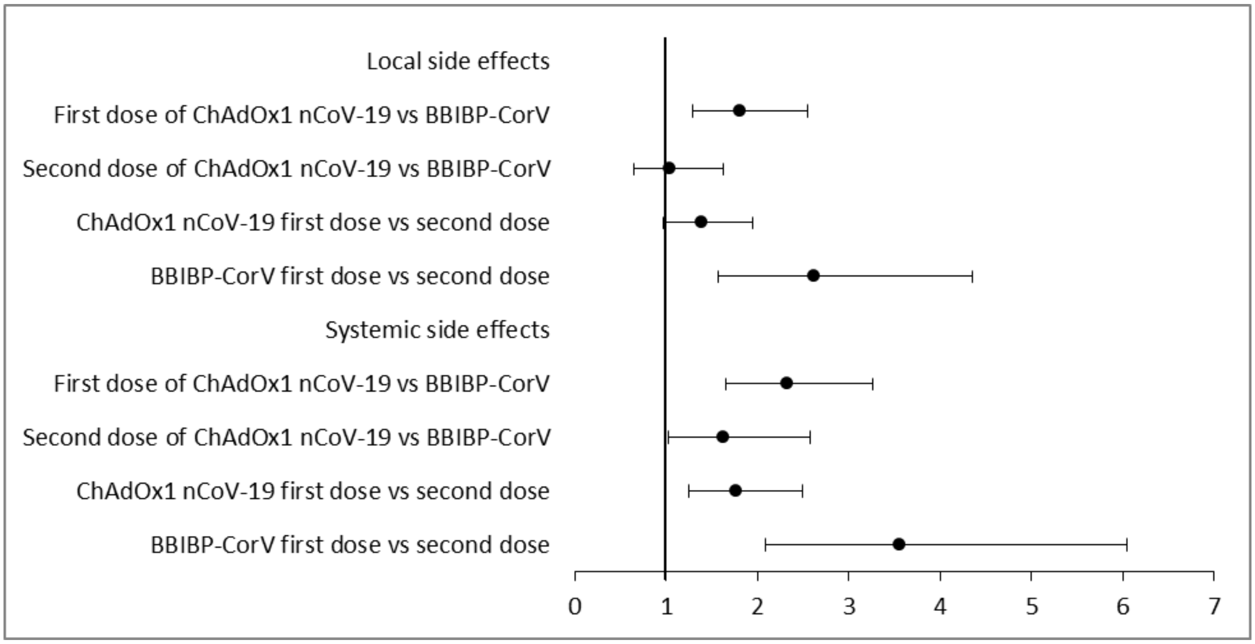

Figure 1: Risk of adverse events according to vaccine type and doses (OR with 95% CI adjusted for age, sex, comorbidities, and previous SARS-CoV-2 infection)

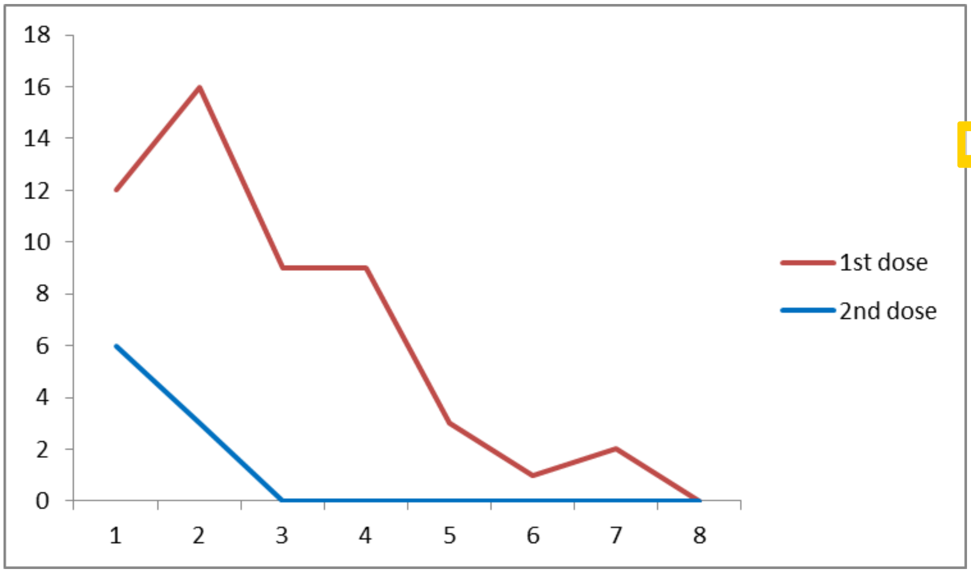

Figure 2: SARS-CoV-2 infection among the participants up to 8 weeks after vaccination (n = 61)

Click here to access/download
Supporting Information
Suplimantary figure.docx
