# Supplementary material for: Adverse events following immunization with ChAdOx1 nCoV-19 and BBIBP-CorV vaccine: A comparative study among healthcare professionals of Nepal
Source: PLoS One. 2022 Aug 10;17(8):e0272729. doi: 10.1371/journal.pone.0272729 (PMC9365127; doi:10.1371/journal.pone.0272729)
Supplement: Attachment — Submitted filename: Response to Reviewers.docx [file pone.0272729.s003.docx]

**Response to Reviewers**	Date: April 30, 2022

**Paper Title:** Adverse events following immunization with ChAdOx1 nCoV-19 and BBIBP-CorV vaccine: a real-world, comparative study among healthcare professionals of Nepal

**Journal Name:** PLOS ONE
**Paper ID:** PONE-D-21-28369

Dear Editor

Thank you very much for providing reviewers’ insightful remarks on our manuscript. We have made the necessary changes and revised the manuscript accordingly, and detailed point–by–point corrections are given below:

**Review Reports:**

**Reviewer #1:**

Thank you for the invitation to review this manuscript. The manuscript is mostly well-written and presented. The study is interesting and relevant. A few corrections to language may be required at certain places.

**Authors reply:** We would like to thank the reviewer for the kind words. We have revised the grammatical errors according to your suggestion.

My concerns are related to the Methodology of the study. The population of health care professionals in Nepal is large and a sample of 606 health care professionals (HCPs) may not adequately represent this population. The sampling method in this study was a convenience one as stated by the authors. I am not sure that this sample can adequately provide data from the population of HCPs in Nepal. The authors have provided no information about the geographical distribution of the respondents within the country. Does the sample correspond to the population of HCPs in Nepal regarding demographic characteristics? I am not sure that the conclusions as mentioned in the abstract follow from the study results. The conclusions in the main manuscript seem OK. As the denominator is small I am not sure prevalence of adverse effects can be calculated. The findings will be only the prevalence among the study sample, and it will be difficult to generalize it to the general population of HCPs in Nepal.

**Authors reply:** We have calculated the sample size for the present study from a previously published article. However, we agree with you that, the sample size included in the present study might not be representative of the large population of the HCPs in Nepal. Moreover, geographic and ethnic variety was also not considered in the present study which limits the generalizability of the findings. We have mentioned these issues in the limitation section of the manuscript (Page 13, Line 223-227).

*“Moreover, the small sample size of the present study might not be representative of the large population of HCPs in Nepal. Moreover, information about the geographic location and ethnic characteristics of the participants were not collected. Hence, the prevalence of vaccine-related adverse events found in the present study might not be generalizable for the overall population of the country.”*

In the Results can the authors explain why they mention no vaccine breakthrough infection was noted.

**Authors reply:** Thanks for mentioning the issue. We have added the definition of vaccine breakthrough infection as an explanation of the statement which was missing in the previous version (Page 10, Line 174-175).

*“No vaccine breakthrough infection which is defined as infection by SARS-CoV-2 after at least 14 days of completion of two-dose vaccination was reported in the study.”*

The authors have not mentioned about IRB approval and obtaining written, informed consent from the participants in this study. I would recommend specialised statistical review of this manuscript.

**Authors reply:** Thanks for mentioning the issue. The statement regarding ethical considerations is now mentioned in the manuscript (Page 6. Line 122-125).

*“The ethical clearance of the research proposal was obtained from the Nepal Health Research Council (NHRC) (Reference no. 351). Informed written consent was obtained through email from the participants who agreed to participate in the study as explained on the first page of the survey form.”*

The authors should deposit the de-identified data in a publicly available repository.

**Authors reply:** Thanks for your suggestion. The data will be made publicly available.

**Reviewer #2:**

The findings from this study will be of importance to advocate the public on vaccination and reduce their anxiety regarding the side effects.

Overall, please check the grammatical errors and paraphrasing needs to be done as per recommendation.

**Authors reply:** We would like to thank the reviewer for the kind words. We have revised the grammatical errors according to your suggestion.

**Results**

1) The first Chi-square analysis seems unnecessary as the parameters are not associated with the dosing completion since the vaccination is dependent upon availability and the set duration between first and second dose.

**Authors reply:** Thanks for your suggestion. We have omitted the Chi-square analysis from Table 1.

2) The graphs need legends and appropriate choice of graph type is imperative.

**Authors reply:** Thanks for your suggestion. We have added legends in the graphs.

**Conclusion**

This article has been clearly written however the conclusion about the anaphylaxis being rare is a bit of a stretch as this has not been evaluated in this study.

**Authors reply:** Thanks for your valuable comment. We agree with you and deleted the statement from the conclusion (Page 13, Line 235).

We would like to thank the reviewers for the valuable comments. We have revised the documents to the best of our ability, but we will definitely be happy to provide further improvement if there are further clarifications required.

With best regards

Dr. Md. Golam Hossain

Professor of Health Research Group

Department of Statistics, University of Rajshahi

Rajshahi-6205, Bangladesh

E-mail: hossain95@yahoo.com
