# Supplementary material for: Adverse events following immunization with ChAdOx1 nCoV-19 and BBIBP-CorV vaccine: A comparative study among healthcare professionals of Nepal
Source: PLoS One. 2022 Aug 10;17(8):e0272729. doi: 10.1371/journal.pone.0272729 (PMC9365127; doi:10.1371/journal.pone.0272729)
Supplement: Attachment — Submitted filename: Response to Reviewers.docx [file pone.0272729.s004.docx]

**Response to Reviewers**	Date: July 23, 2022

**Paper Title (old):** “Adverse events following immunization with ChAdOx1 nCoV-19 and BBIBP-CorV vaccine: a real-world, comparative study among healthcare professionals of Nepal”

**Paper title (New):** “Adverse events following immunization with ChAdOx1 nCoV-19 and BBIBP-CorV vaccine: a comparative study among healthcare professionals of Nepal”

**Journal Name:** PLOS ONE
**Paper ID:** PONE-D-21-28369R1

Dear Editor

Thank you very much for providing reviewers’ insightful remarks on our manuscript. We have made the necessary changes and revised the manuscript accordingly, and detailed point–by–point corrections are given below:

**Review Reports:**

**Reviewer #1:**

Kindly deposit the data associated with the study in a public repository. Otherwise the authors have addressed the comments.

**Authors’ reply:** Thank you for your comment. We have already submitted the dataset to the Journal repository during manuscript submission.

**Reviewer #3:**

**Title:**
I suggest removing the title of "Real world". The instrument in this case was designed specifically for this study.

**Authors’ reply:** Thank you for your suggestion. According to your suggestion, we have removed “Real world” from the title. The new title is, “Adverse events following immunization with ChAdOx1 nCoV-19 and BBIBP-CorV vaccine: a comparative study among healthcare professionals of Nepal”
**Summary:**
Avoiding the term "fully", currently there are people with 3 or even 4 doses of vaccination.

**Authors’ reply:** We have avoided the term “fully” [Line: 46].  **Introduction:**
I suggest considering an additional base bibliography. https://doi.org/10.54034/mic.e1262

**Authors’ reply:** We have added the suggested bibliography [reference 4].

**Methods:**
There was some way to confirm that the respondents were health personnel. The instrument was online, so how could membership in the group of HCWs be verified?

**Authors’ reply:** Thank you for your comment. We have circulated the online questionnaire within the professional social media groups of the HCWs. However, we did not take any specific measure to verify their identity as HCWs which we have mentioned in the limitation section [Page 13, line 234-235].
**Results.**
Avoid the use of "partially" and "Fully". Indicate 1 or two doses.
Were there subjects with a heterologous vaccine schedule?

**Authors’ reply:** Thank you for the suggestion. We have corrected the manuscript accordingly [Table 1, Page, 7; Line:138-141].

**Discussion.**
Add a paragraph about the possible presence of variants during the study period. <https://doi.org/10.54034/mic.e1256>

**Authors’ reply:** Thank you for the suggestion. We have added the statement in the discussion [Page 12, line 215-216]

We would like to thank the reviewers for the valuable comments. We have revised the documents to the best of our ability, but we will definitely be happy to provide further improvement if there are further clarifications required.

With best regards

Dr. Md. Golam Hossain

Professor of Health Research Group

Department of Statistics, University of Rajshahi

Rajshahi-6205, Bangladesh

E-mail: hossain95@yahoo.com
